# Identification of biomarkers for the diagnosis of chronic kidney disease (CKD) with dilated cardiomyopathy (DCM) by bioinformatics analysis and machine learning

**DOI:** 10.3389/fgene.2025.1562891

**Published:** 2025-05-30

**Authors:** Yuhang Liu, Yong Wang, Wenyang Nie, Zhen Wang

**Affiliations:** ^1^ College of First Clinical Medicine, Shandong University of Traditional Chinese Medicine, Jinan, China; ^2^ Department of Cardiology, Affiliated Hospital of Shandong University of Traditional Chinese Medicine, Jinan, China

**Keywords:** dilated cardiomyopathy, chronic kidney disease, machine learning, WGCNA, immune infiltration, single-cell sequencing

## Abstract

**Background:**

Chronic kidney disease (CKD) is a globally prevalent and highly lethal condition, often accompanied by dilated cardiomyopathy (DCM), which increases the risk of cardiac complications. Early detection of DCM in CKD patients remains challenging, despite established research demonstrating the relationship between CKD and cardiac abnormalities.

**Methods:**

We retrieved expression matrices for DCM (GSE57338, GSE29819) and CKD (GSE104954) from GEO and a DCM scRNA-seq dataset (GSE145154). These were analyzed for differential gene expression and WGCNA. KEGG and GO analyses were performed on shared differentially expressed genes in DCM and CKD. Potential drugs for DCM were identified using CMAP. Machine learning methods LASSO, SVM-RFE, and RF were used to find biomarkers and develop a diagnostic nomogram for CKD-associated DCM, validated with external datasets. Single-gene GSEA was conducted to understand model gene mechanisms in CKD-associated DCM. Immune cell infiltration was analyzed with CIBERSORT, and single-cell sequencing examined model gene distribution and expression in the heart.

**Results:**

Our examination of the expression matrix datasets associated with DCM and CKD revealed 115 key model genes that are shared by the two disorders as well as 47 genes that are differently expressed. These 47 differentially expressed genes were primarily linked to immune regulation and inflammation, according to enrichment analysis. CMAP analysis suggested withaferin-a, droxinostat, fluorometholone, and others as potential DCM treatments. Machine learning identified MNS1 and HERC6 as significant CKD-associated DCM biomarkers. A diagnostic nomogram using these genes was developed, showing strong discriminative power and clinical utility. MNS1 and HERC6 are implicated in metabolism, inflammation, immunity, and heart function. Immune cell infiltration analysis indicated dysregulation in DCM, with MNS1 and HERC6 correlating with immune cells. Single-cell sequencing showed MNS1 and HERC6 expression in endothelial cells and fibroblasts, respectively.

**Conclusion:**

We identified MNS1 and HERC6 as biomarkers and developed a new diagnostic nomogram based on them for the timely diagnosis of CKD patients presenting with DCM complications. This study’s findings offer novel insights into potential diagnostic methods and therapeutic strategies regarding the coexistence of CKD and DCM.

## 1 Introduction

Chronic Kidney Disease (CKD) significantly contributes to morbidity and mortality associated with non-communicable diseases. It is characterized by a structural or functional kidney abnormality that persists for over 3 months, primarily involving tubular, glomerular, or interstitial damage. This condition impairs the kidneys’ ability to effectively eliminate waste products and excess water from the body. Assessment of CKD involves various parameters, including glomerular filtration rate (GFR), the threshold for proteinuria, and the duration of the injury (Webster et al., 2017). Screening conducted in various countries during the 2000s revealed that indicators of kidney disease are evident in over 10% of adults. As of 2017, the global prevalence of CKD rose to 9.1%, reflecting a 29.3% increase since 1990 (Bikbov et al., 2020). This trend indicates a significant escalation in the burden of CKD, presenting a formidable public health challenge globally in terms of effective management (Lin et al., 2022).

Dilated cardiomyopathy (DCM) is a progressive cardiac condition that arises mainly without abnormal loading conditions or significant coronary artery disease. It is characterized by ventricular dilation, thinning of the ventricular walls, and impaired dilation and contraction of the left ventricle or both ventricles, leading to compromised cardiac pumping function (Japp et al., 2016; Heymans et al., 2023). Prior research has established a robust correlation between CKD and modifications in heart structure and function, particularly in CKD patients, for whom cardiovascular illness is a significant contributor to morbidity and mortality (Di Lullo et al., 2015a; Thomas et al., 2017).In the setting of CKD, 80% of individuals are classified as being at elevated risk for cardiovascular events, with significant cardiac incidents accounting for over 50% of mortality causes among those with CKD (Heywood et al., 2007; Anand et al., 2015). The significant cardiac burden in the CKD population frequently results in dilated cardiomyopathy, which ultimately leads to the clinical manifestations of heart failure (HF) and may exert a more severe prognostic influence than the symptoms alone (Curtis and Parfrey, 2005).

Inadequate diagnosis and intervention in DCM complicate the reversal of the disease after it has advanced to serious cardiac problems; prompt therapies are essential for enhancing the prognosis of CKD-associated DCM. Nonetheless, we continue to encounter numerous obstacles in early diagnosis. Despite the widespread use of endomyocardial biopsy techniques, the precise detection of myocardial injury linked to CKD continues to pose a difficulty due to its poor sensitivity (Cooper et al., 2007). Consequently, the status of the majority of DCM patients typically deteriorates steadily, ultimately resulting in severe heart failure or mortality. Furthermore, from the standpoint of pathological mechanisms, CKD may negatively influence the structure and function of the heart via multiple routes, including, but not limited to, inflammation and oxidative stress (Di Lullo et al., 2015b). The co-morbid mechanisms entail complex interactions, and the principal molecules and underlying processes remain unspecified; thus, there is an imperative to identify potential biomarkers, establish a more comprehensive diagnostic model, and investigate the associated mechanisms for the early diagnosis and intervention of CKD-associated DCM.

The rapid advancement of microarray and high-throughput sequencing technologies has significantly enhanced the reliability and persuasiveness of bioinformatics analyses. In this study, gene expression matrices from two DCMs and one CKD, sourced from the GEO database, were employed alongside a single-cell RNA sequencing (scRNA-seq) dataset related to DCM. Integrated bioinformatics methods were utilized to identify hub genes and potential mechanisms linking CKD to DCM. A diagnostic nomogram, incorporating the genes MNS1 and HERC6, was developed for DCM using machine learning techniques. The validity of this nomogram was subsequently assessed and confirmed using external datasets. Furthermore, the study explored the relationship between the two model genes and the immunological landscape, utilizing single-cell sequencing to reveal the expression distribution of these genes across various cardiac cell types.

## 2 Methods

### 2.1 Data acquisition and preliminary processing

**FIGURE 1:**
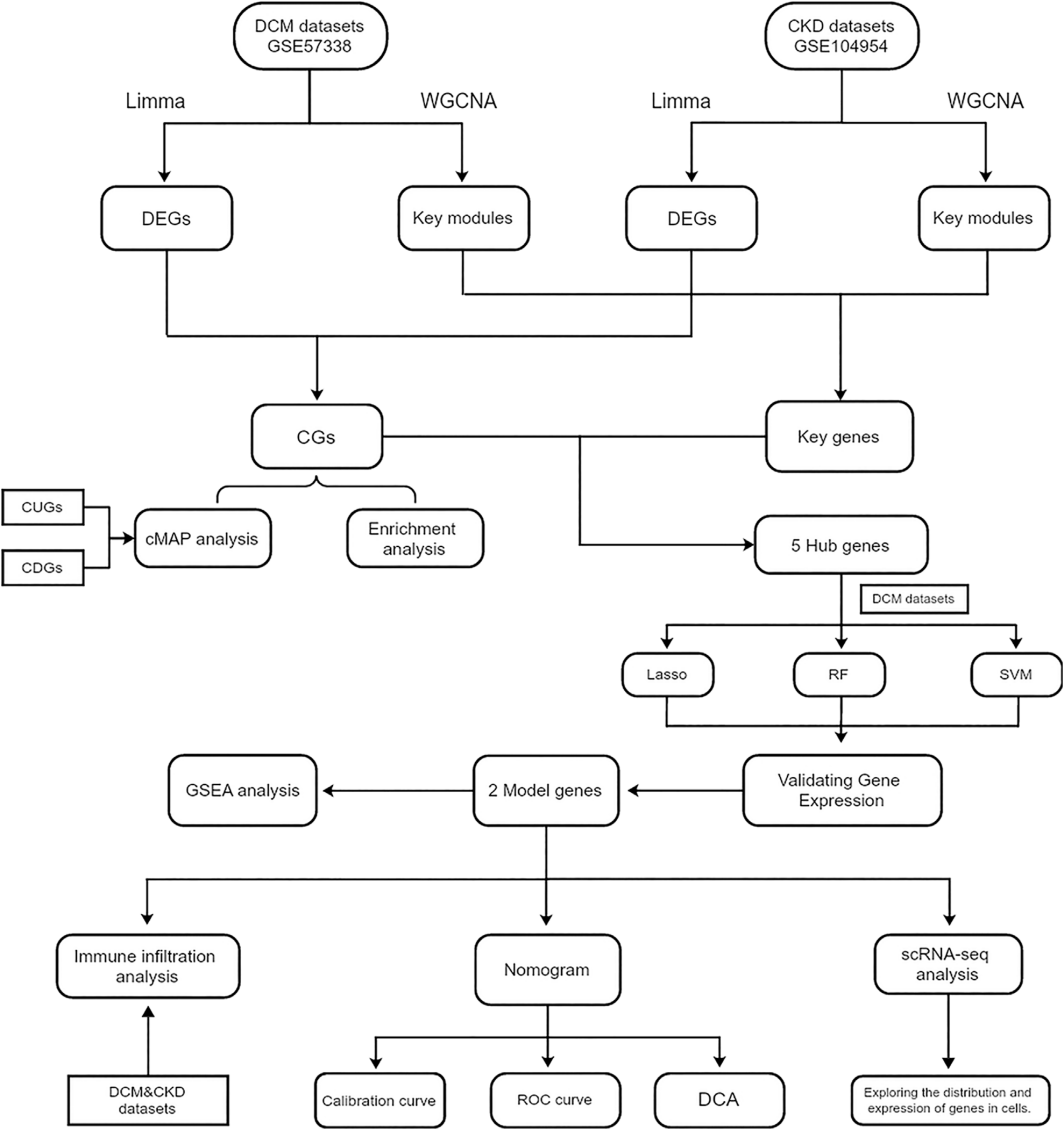
Flowchart of the steps of bioinformatics analysis. Abbreviations: CKD, Chronic Kidney Disease; DCM, Dilated Cardiomyopathy; WGCNA, Weighted gene co-expression network analysis. DEGs, Differentially expressed genes; CGs, Common genes; CUGs, Co-upregulated genes; CDGs, Co-downregulated genes; ROC, Receiver operating characteristic; DCA, Decision curve analysis.

The study utilized two raw expression profile datasets for DCM, specifically GSE57338 and GSE29819, as well as one dataset for CKD, GSE104954, all of which were sourced from the GEO database (https://www.ncbi.nlm.nih.gov/geo/). To convert probe matrices into gene matrices, the “GEOquery” package in R was employed, utilizing probe annotation files during the conversion process. For genes represented by multiple probes, expression values were averaged across their corresponding probes. Additionally, a single-cell RNA sequencing dataset related to DCM, identified as GSE145154, was obtained from the same database. Table 1 provides comprehensive information on all training and external validation sets that form the foundation of this study. The workflow diagram is depicted in Figure 1.

**TABLE 1:** Details of the 4 GEO datasets.

Data sets	Platform	Type of samples	Control sample size	CKD or DCM sample size	Applications
GSE57338	GPL11532	DCM	136	82	Screening DEGs and Key modules; Training of hub genes
GSE29819	GPL570	DCM	12	14	Validation of hub genes
GSE104954	GPL22945	CKD	18	39	Screening DEGs and Key modules; Training of hub genes
GSE145154	GPL20795	DCM	4	8	Single-cell sequencing data analysis

### 2.2 Differentially expressed genes (DEGs) analysis

This analysis employs the “Limma” package within the R software (Ritchie et al., 2015) to identify differentially expressed genes (DEGs) in the CKD and DCM datasets (GSE57338 and GSE104954). The criteria for identifying DEGs were set at a p-value of less than 0.05 and an absolute log2 fold change greater than 0.585 (Udhaya Kumar et al., 2020; Alva et al., 2022; Zhang et al., 2023). To visualize the expression patterns of these DEGs, the “pheatmap” and “ggplot2” packages in R were utilized. Consequently, volcano plots were generated to represent all DEGs, while clustered heat maps were created to display the top 100 upregulated and top 100 downregulated genes.

### 2.3 Weighted gene co-expression network analysis and identification of key model genes

Weighted Gene Co-expression Network Analysis (WGCNA) was implemented to systematically identify co-expressed gene modules, elucidate the relationships between gene networks and phenotypes, and determine key regulatory genes within these networks. The analysis was performed using the “WGCNA” package in R software (Langfelder and Horvath, 2008), which enabled the construction of comprehensive co-expression networks encompassing all genes in the dataset. Network connectivity was assessed through the transformation of the weighted adjacency matrix into a topological overlap matrix (TOM), from which a hierarchical clustering tree structure was derived. The resulting dendrogram branches represented distinct gene modules, differentiated by color coding, with genes exhibiting similar expression patterns and functional characteristics clustered within the same module. To optimize the network structure, modules with high similarity were merged using a “mergeCutHeight” threshold of 0.25 (Dobnikar et al., 2018; Liu et al., 2023b).

### 2.4 GO and KEGG functional analysis

To explore the biological functions and pathogenic mechanisms of common differentially expressed genes in CKD and DCM, we performed comprehensive GO and KEGG pathway enrichment analyses. The analyses were conducted using multiple packages, including “org.Hs.eg.db,” “GOplot,” “enrichplot,” and “clusterProfiler.”. Statistical significance for enrichment analysis was set at p < 0.05. The results of functional enrichment analysis were visualized through various graphical representations, including circle and composition plots, generated using “ggplot2” and OmicShare Tools (Mu et al., 2024).

### 2.5 Connectivity map analysis

The Connectivity Map (CMAP) database (https://clue.io) serves as a comprehensive repository for gene expression profiling data, enabling researchers to investigate the impact of various perturbations on gene expression patterns and elucidate the complex relationships between genes and small molecules. In our investigation, we leveraged this database by analyzing DEGs that exhibited consistent up- or downregulation patterns in both DCM and CKD. This analytical approach was specifically designed to identify potential therapeutic compounds for DCM treatment. Through systematic analysis, we identified and ranked the top 10 compounds based on their enrichment scores. To facilitate the interpretation of these findings, we constructed a Sankey diagram using the “ggalluvial” R package, which effectively visualized the relationships and characteristic properties of the identified compounds.

### 2.6 Machine learning

Three machine learning algorithms—Least Absolute Shrinkage and Selection Operator (LASSO), Support Vector Machine Recursive Feature Elimination (SVM-RFE), and Random Forest (RF)—were employed to refine the selection of candidate biomarkers. First, our study implemented the “randomForest” package to perform the RF algorithm, and the hub gene was defined as MeanDecreaseGini (MDG) greater than 4.0 (Savargiv et al., 2021; Walker et al., 2022). Subsequently, we conducted LASSO regression analysis utilizing the “glmnet” software package. The optimal λ value in the LASSO model, characterized by the lowest mean square error, was identified, and the genes exhibiting good predictive capability associated with this λ value were picked (Friedman et al., 2010) SVM-RFE for narrowing down candidate biomarkers for the “e1071” package (Meyer et al., 2020).

To mitigate overfitting—particularly in the context of limited sample sizes—we employed five-fold cross-validation across all three algorithms. LASSO utilized L1 regularization to penalize overly complex models by shrinking uninformative coefficients to zero. SVM-RFE integrated recursive feature elimination with repeated cross-validation to identify stable and discriminative features. For Random Forest, out-of-bag (OOB) error estimation was applied, and the number of trees was selected based on the lowest classification error. These strategies collectively enhanced the robustness of the gene selection process.

The intersecting genes identified by the three machine learning techniques were designated as hub genes for the formulation of diagnostic models for CKD-associated DCM. The expression levels of the hub genes were determined using two datasets, GSE57338 and GSE104954.Finally, the genes with common expression trends were selected as the model genes for subsequent analysis.

### 2.7 Construction of column line diagrams and evaluation of predictive models for diagnostic markers

To evaluate the model genes, we first performed multivariate logistic regression analyses. Subsequently, we constructed a diagnostic nomogram using the “rms” package. The predictive performance of both individual model genes and the integrated diagnostic model was assessed through ROC analysis implemented via the “ROCR” package. To validate the clinical utility and predictive accuracy of the nomogram, we employed Decision Curve Analysis (DCA) and calibration curves, which were generated using the “calibration” function from the “rmda” package and the “rms” package, respectively. The robustness of our findings was further verified using GSE29819 as an external validation cohort.

### 2.8 Single gene GSEA analysis

To explore the potential enrichment of model genes in various biological processes, we conducted single-gene Gene Set Enrichment Analysis (GSEA). Initially, model genes were sequenced based on their correlation with other genes. Subsequently, these genes underwent GSEA using the GSE57338 expression profiles. The analysis focused on identifying relevant pathways and biological processes, utilizing the Molecular Signatures Database with Gene Ontology as the background gene set. Statistical significance was determined by a |normalized enrichment score (NES)| greater than 0.5 and an adjusted P-value of less than 0.05.

### 2.9 Analysis of immune infiltration

The “CIBERSORT” package was utilized to evaluate immune cell infiltration in the gene expression profiles of DCM and CKD. The quantity and ratio of immunological infiltration in each sample were depicted as bar graphs 。utilizing the “ggplot2” software. The proportions of 22 immune cell types were examined between DCM and normal samples. The results are presented through stacked histograms produced by the “ggplot2” software. The relationship between the two model genes and the differential immune cells was analyzed using the “ggplot2” software, with a significance threshold of p < 0.05 deemed statistically significant.

### 2.10 Assessing model gene expression on single-cell RNA-seq data

The data were initially processed using the Seurat software package (Stuart et al., 2019), followed by analysis with the UMAP method to identify the interrelationships between clusters and cell types. Subsequently, pertinent marker genes were acquired, and cells were primarily annotated by referencing the Cell Marker 2.0 database (http://yikedaxue.slwshop.cn/) (Zhang et al., 2019). A threshold was applied to marker gene expression levels, specifically min.pct = 0.25 and log2FC > 1, to ensure robust cell type identification.

### 2.11 Statistical analysis

Statistical analyses were performed using the R programming language (version 4.4.0). The diagnostic performance of each model gene was evaluated through the AUC score. A p-value below 0.05 was considered statistically significant.

## 3 Results

### 3.1 Weighted gene co-expression network analysis and characterization of key module genes

**FIGURE 2:**
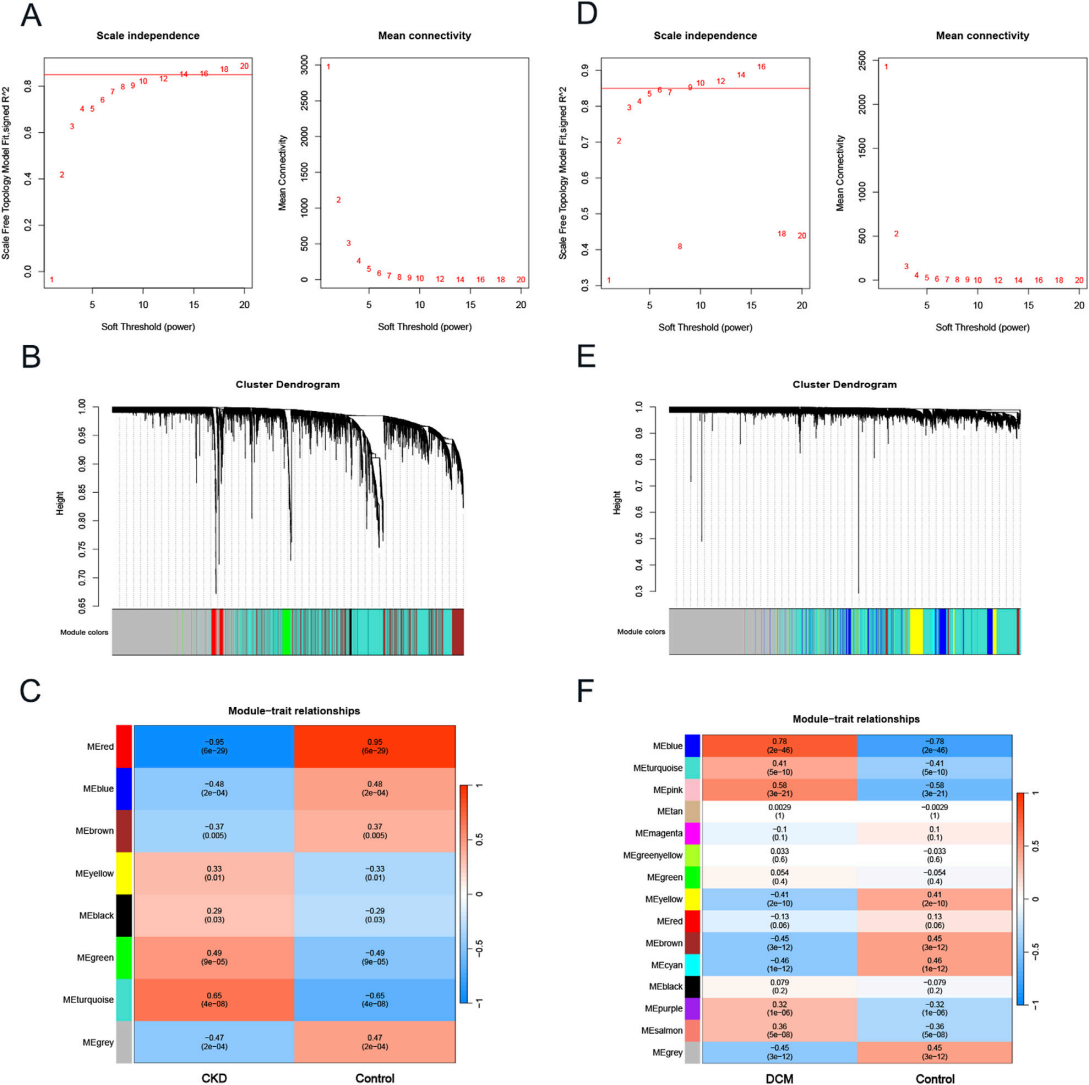
The construction of weighted gene co-expression network in CKD and DCM. **(A)** β = 14 was selected as the soft threshold for CKD, based on scale independence and average connectivity. **(B)** Dendrogram of gene clustering in CKD, with different colors representing different modules. **(C)** Correlation between module eigengenes and CKD, where blue indicates negative correlation and red indicates positive correlation. **(D)** β = 9 was selected as the soft threshold for DCM, based on scale independence and average connectivity. **(E)** Dendrogram of gene clustering in DCM, with different colors representing different modules. **(F)** Correlation between module eigengenes and DCM, where blue indicates a negative correlation and red indicates a positive correlation. Abbreviations: CKD, Chronic Kidney Disease; DCM, Dilated Cardiomyopathy; WGCNA, weighted gene co-expression network analysis.

To investigate key genes associated with CKD and DCM, we conducted WGCNA to identify the most relevant gene modules in samples from both conditions. In the CKD-WGCNA analysis, a soft threshold of 14 was selected based on scale independence and average connectivity, optimizing the performance of scale-free networks (scale-free *R* ^2^ = 0.85; Figure 2A). The dendrogram illustrating module clustering is presented in Figure 2B. Our analysis revealed that the turquoise module, comprising 2376 genes, exhibited the most significant positive correlation with CKD (r = 0.65, p = 4e-08), thereby identifying it as a key module for further investigation (Figure 2C). Similarly, in the DCM-WGCNA analysis, a soft threshold of 9 was chosen (Figure 2D), and the corresponding dendrogram of module clustering is shown in Figure 2E. The blue module, containing 866 genes, demonstrated the strongest positive correlation with DCM (r = 0.78, p = 2e-46), designating it as a key module for subsequent study (Figure 2F). Following this, we intersected the key module genes identified in the CKD and DCM datasets to pinpoint 115 important genes, referred to as Key genes, for further investigation (Supplementary Figure S1).

### 3.2 Screening of differentially expressed genes (DEGs)

**FIGURE 3:**
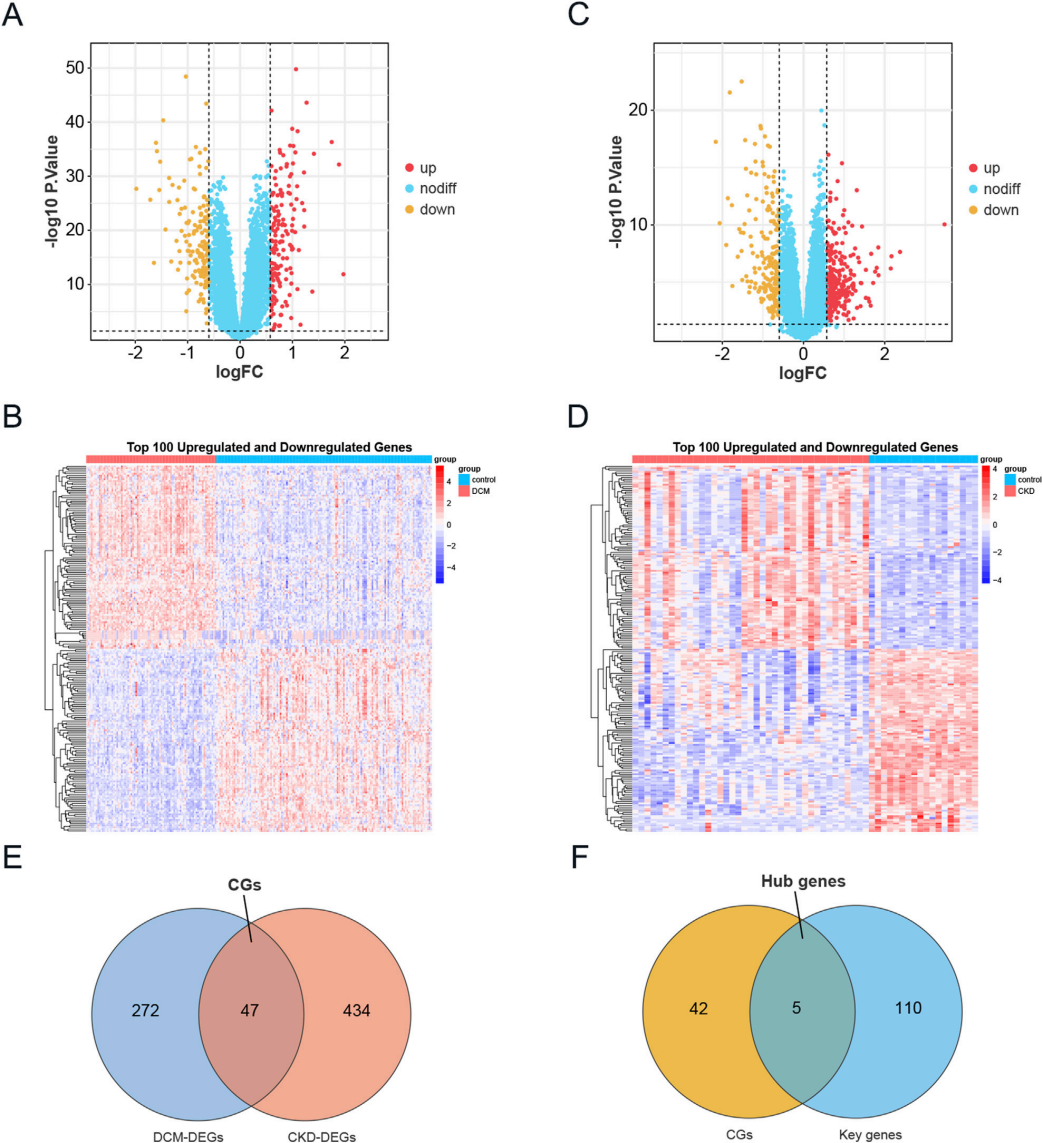
Identification of DEGs in CKD and DCM. **(A)** The volcano plot of DEGs in DCM dataset GSE57338. Red and yellow indicate the most significantly upregulated and downregulated differentially expressed genes in the DCM samples, respectively. **(B)** Heatmap of the top 100 upregulated and 100 downregulated DEGs in the DCM dataset. **(C)** The volcano plot of DEGs in CKD dataset GSE104954. Red and yellow indicate the most significantly upregulated and downregulated differentially expressed genes in the CKD samples, respectively. **(D)** Heatmap of the top 100 upregulated and 100 downregulated DEGs in the CKD dataset. **(E)** Venn diagram showing the intersection of DEGs in DCM and CKD, named CGs. **(F)** A Venn diagram was used to identify Hub genes by intersecting the key genes from the WGCNA modules in DCM and CKD with CGs. Abbreviations: CKD, Chronic Kidney Disease; DCM, Dilated Cardiomyopathy; DEGs, Differentially expressed genes; CGs, Common genes.

A differential analysis comparing CKD samples to normal samples identified a total of 481 DEGs, with 282 genes upregulated and 199 downregulated. In contrast, DCM revealed 319 DEGs, consisting of 160 upregulated and 159 downregulated genes. The expression profiles of these DEGs in both the CKD and DCM datasets were visually represented using volcano plots and heat maps, as shown in Figures 3A,B,D,E. By intersecting the DEGs from CKD and DCM samples using the “VennDiagram” package in R software, we identified 47 common genes (CGs), as depicted in Figure 3C. DEGs and CGs are shown in Supplementary File S1. Additionally, by cross-referencing these CGs with 115 previously recognized key genes, we identified 5 hub genes, as illustrated in Figure 3F.

### 3.3 Functional enrichment analysis

**FIGURE 4:**
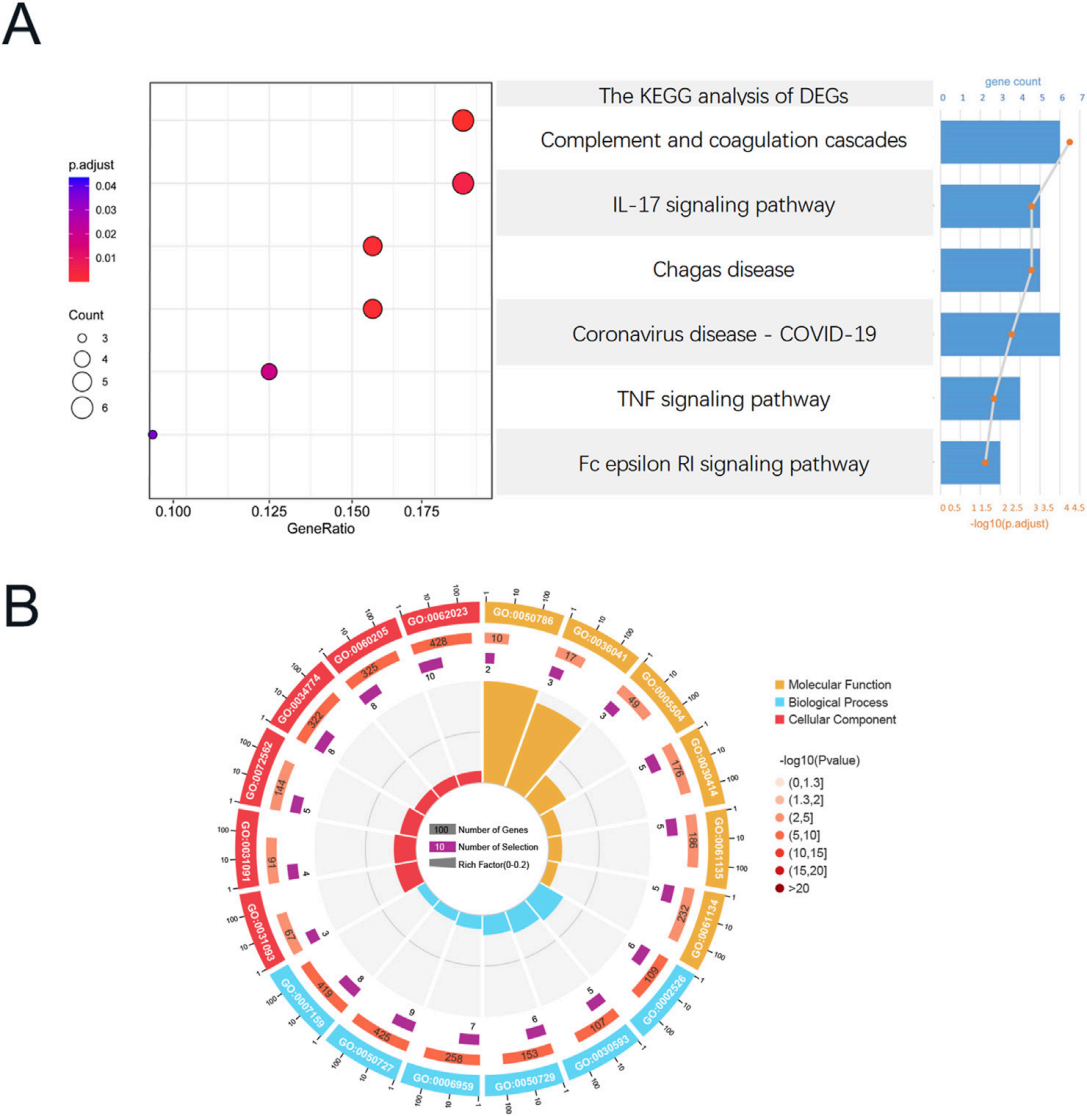
Functional enrichment analysis of CGs, Functional enrichment analysis of CGs. **(A)** Composition plot of the results of KEGG enrichment analysis based on CGs. **(B)** Circular plot of the results of GO enrichment analysis based on CGs. Abbreviations: CGs, Common genes; GO, Gene ontology; KEGG, Kyoto Encyclopedia of Genes and Genomes.

Functional analysis was conducted based on the acquired CGs to ascertain the probable mechanism of CKD-associated DCM. KEGG enrichment analysis indicated that these genes were considerably abundant in the Complement and coagulation cascades, IL-17 signaling pathway, TNF signaling pathway, and Fc epsilon RI signaling pathway (Figure 4A). Figure 4B illustrates that the Gene Ontology enrichment analysis of these genes corroborated the participation of immunological and inflammatory responses in the pathophysiological mechanisms of CKD-associated DCM, particularly highlighting “neutrophil chemotaxis” and “leukocyte cell-cell adhesion.” In the Gene Ontology analysis of biological processes (BP), pathogenic genes associated with CKD-associated DCM were predominantly enriched in the categories of “regulation of inflammatory response,” “humoral immune response,” “inflammatory response,” “immune response,” “neutrophil chemotaxis,” and “leukocyte cell-cell adhesion.” The Gene Ontology analysis of cellular components (CC) indicated that these pathogenic genes are predominantly situated in the “blood microparticle,” particularly inside the “platelet alpha granule,” “cytoplasmic vesicle lumen,” “secretory granule lumen,” and “collagen-containing extracellular matrix.” Molecular function (MF) study indicated that “RAGE receptor binding” and “long-chain fatty acid binding” were the most pertinent functionalities in the pathogenic genes. The integrated analysis revealed that the causal genes and possible mechanisms of CKD-associated DCM were markedly enriched in inflammatory and immune response pathways. For comprehensive GO analysis results, please consult Supplementary File S2.

### 3.4 Identification of prospective small molecule agents for the treatment of DCM

**FIGURE 5:**
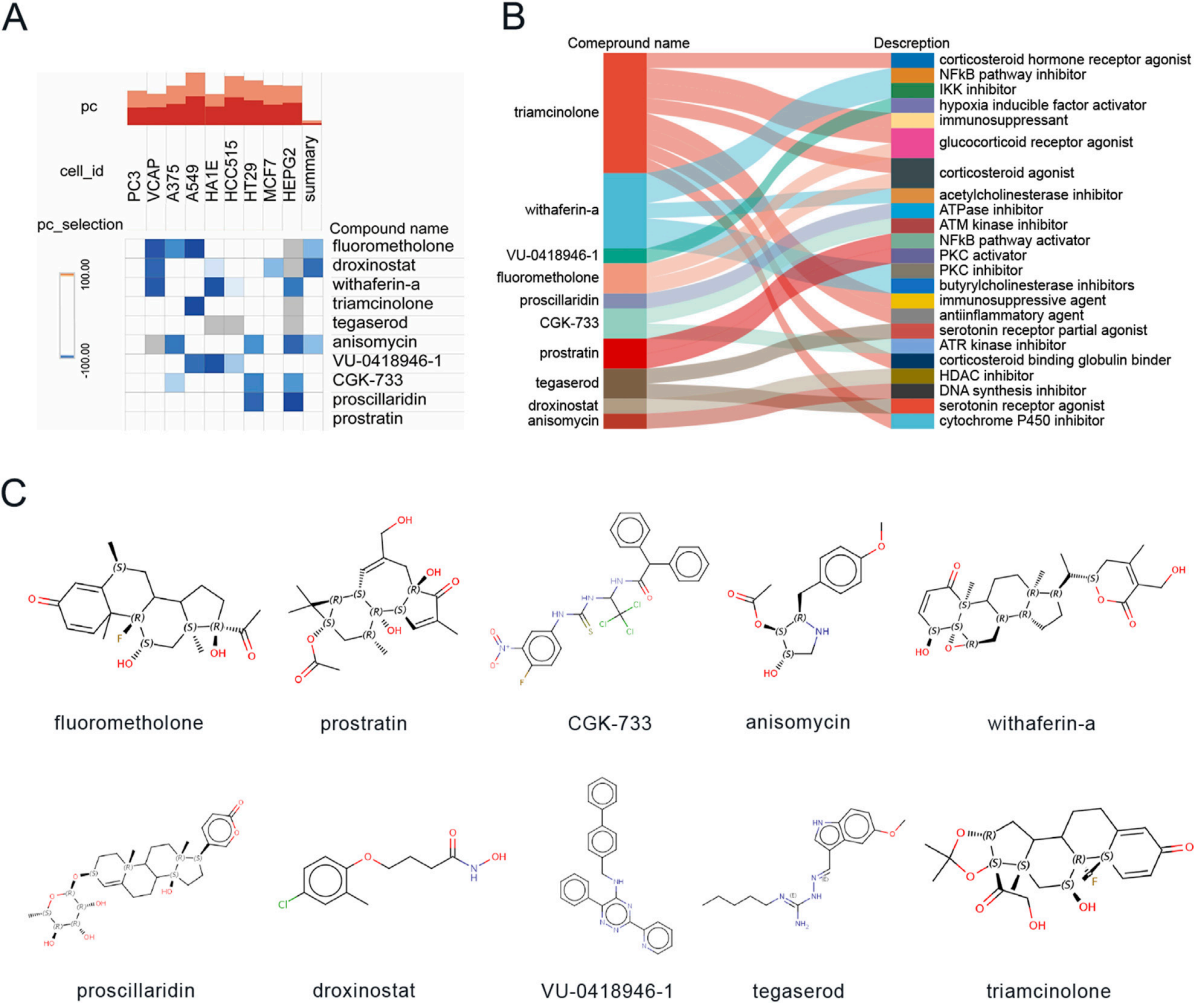
Screening of potential small molecule compounds for DCM treatment using connectivity map analysis. **(A)** Heatmap displaying the top 10 compounds with the highest enrichment scores across 9 cell lines based on connectivity map analysis. **(B)** Descriptions of the top 10 compounds. **(C)** Chemical structures of these 10 compounds.

To identify potential small molecule therapeutics for CKD-associated DCM patients, we leveraged the CMAP database to screen for compounds capable of reversing disease-specific gene expression signatures. We focused on analyzing DEGs that showed concurrent upregulation or downregulation in both CKD and DCM conditions, with the detailed DEGs data presented in Supplementary File S3. Through computational analysis using the CMAP platform (https://clue.io/query), we generated 8,969 prediction outcomes encompassing 2,429 distinct chemical compounds based on these co-regulated DEGs. Following the exclusion of untargeted medicines, we identified and prioritized ten compounds demonstrating the most significant negative median tau scores, indicating their potential to reverse disease-associated gene expression patterns. These promising therapeutic candidates include triamcinolone, withaferin-a, anisomycin, fluorometholone, droxinostat, tegaserod, proscillaridin, VU-0418946-1, CGK-733, and prostratin (Figure 5A). The molecular targeting mechanisms and chemical structures of these compounds are comprehensively illustrated in Figures 5B,C, respectively, providing insights into their potential therapeutic applications in CKD-associated DCM management.

### 3.5 Identifying hub genes of diagnostic value through machine learning

**FIGURE 6:**
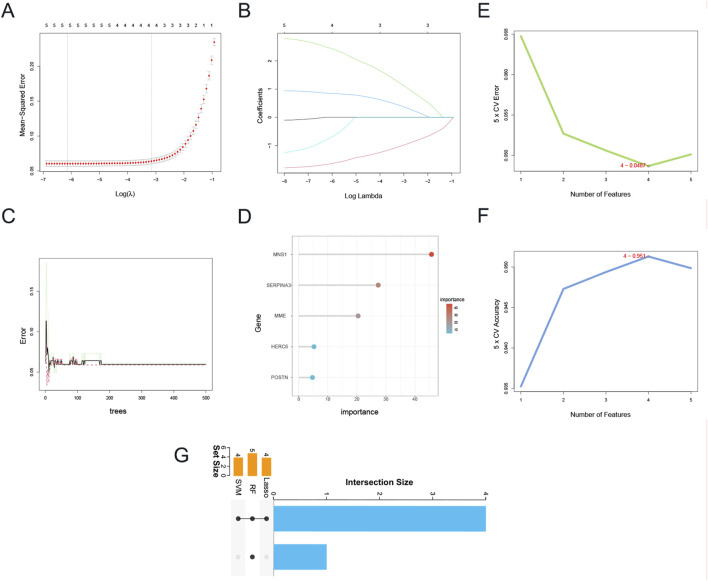
Identification of candidate hub genes via machine learning. **(A)** Plot of partial likelihood deviance. **(B)** Plot of LASSO coefficient profiles. **(C)** The decision tree in RF algorithm. **(D)**The MeanDecreaseGini of the 5 genes with MeanDecreaseGini >1.0 were selected by the RF algorithm. Accuracy **(E)** and error **(F)** of 5-fold cross-validation (CV) in SVM-RFE algorithms. **(G)** UpSet Venn diagram showing the characteristic genes shared by three algorithms. Abbreviations: LASSO, Least Absolute Shrinkage and Selection Operator; RF, Random Forest; SVM-RFE, Support Vector Machine-Recursive Feature Elimination.

Given that DEGs common to CKD and DCM may significantly influence patients with CKD-associated DCM, the LASSO regression algorithm was utilized on five hub genes. The findings indicated that four of these genes could serve as potential candidates with considerable implications for the diagnosis of CKD-associated DCM (Figures 6A,B). Notably, in Figures 6C,D, the MeanDecreaseGini of all five hub genes found by the Random Forest algorithm above 4.0, whereas four genes were identified based on the importance of SVM-RFE calculations and 5-fold cross-validation findings (Figures 6E,F). Ultimately, we intersected the genes identified by the three approaches, resulting in four genes: SERPINA3, MNS1, MME, and HERC6 (Figure 6G).

### 3.6 Validation of four gene expression levels in DCM and CKD

**FIGURE 7:**
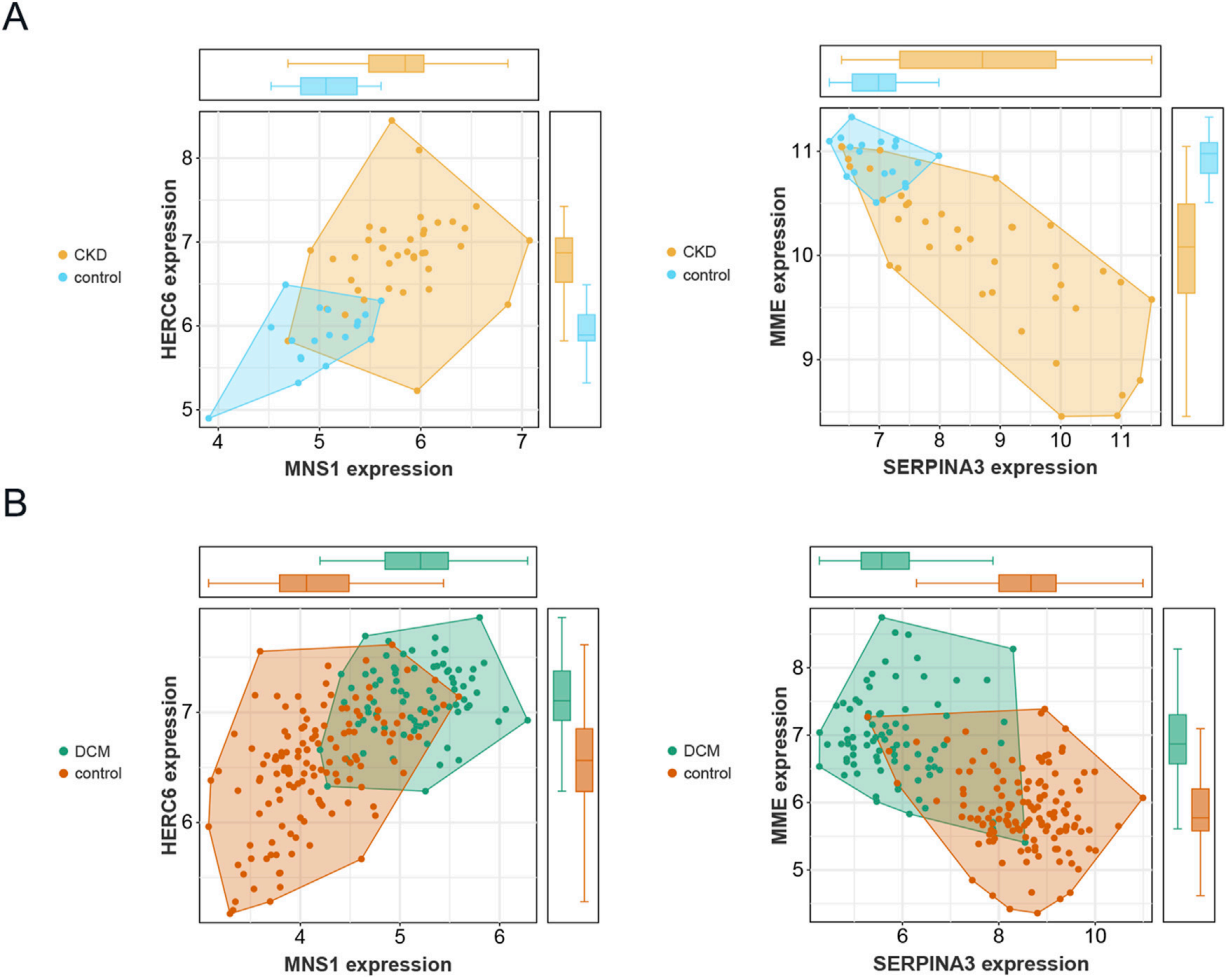
Validation of the expression levels of four genes in CKD and DCM. **(A)** Expression patterns of the 4 genes in the CKD dataset GSE104954. **(B)** Expression patterns of the 4 genes in the DCM dataset GSE57338.

Figure 7A illustrates that in the CKD dataset (GSE104954), the expression levels of MNS1, HERC6, and SERPINA3 were markedly increased in CKD patients relative to controls. Nonetheless, the expression levels of MME were decreased in patients with CKD. In the DCM dataset (GSE57338), as illustrated in Figure 7B, the expression levels of MNS1, HERC6, and MME were markedly elevated in DCM patients relative to controls. Conversely, the expression level of SERPINA3 was reduced in patients with DCM. Consequently, it can be inferred that among the four selected genes, only the expression levels of MNS1 and HERC6 were stable in CKD and DCM patients, both exhibiting upregulation, indicating a link between their gene expression levels and illness development.

### 3.7 Construction of nomogram and evaluation of diagnostic biomarker prediction models

**FIGURE 8:**
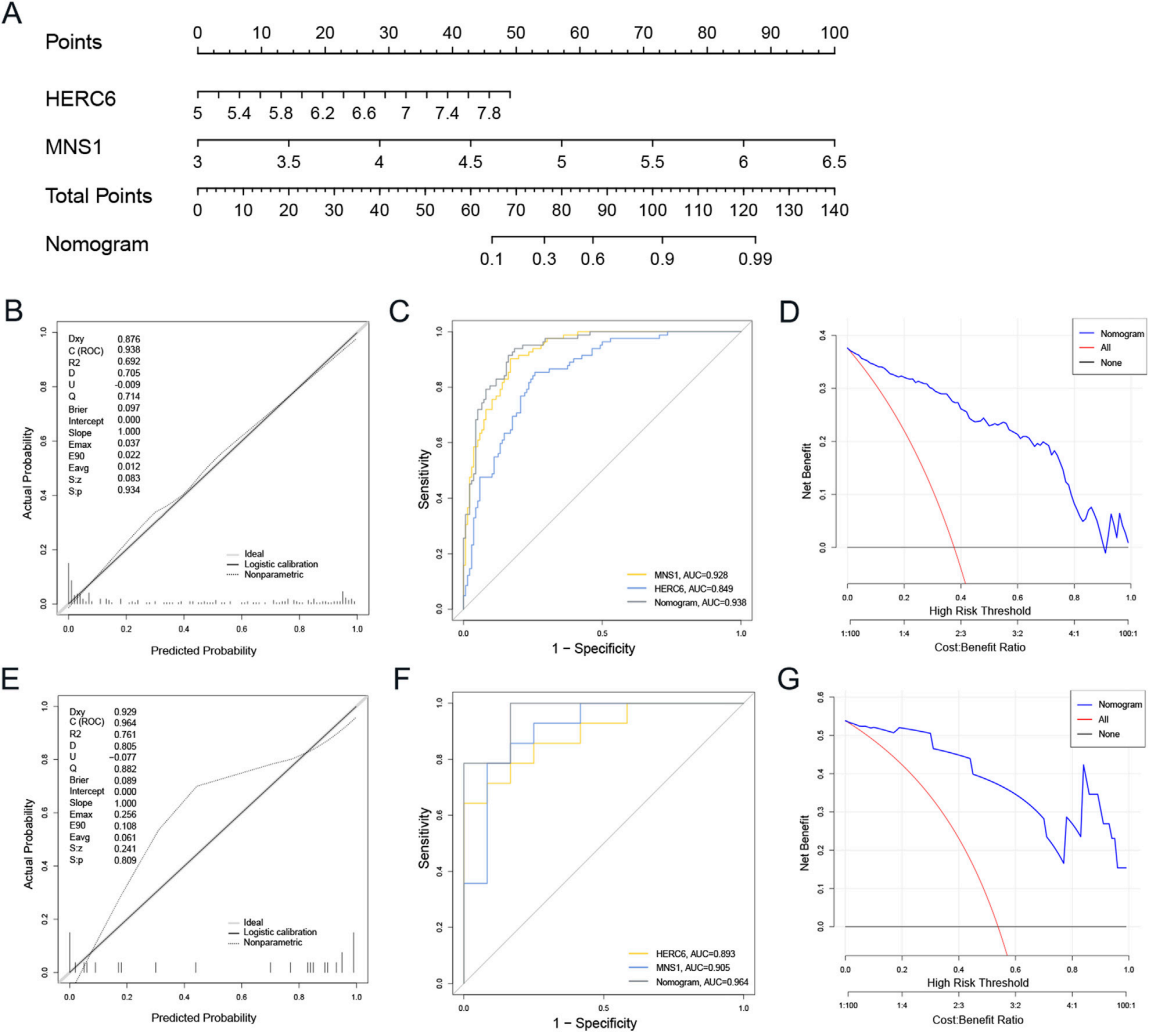
The development and efficacy evaluation of the diagnostic nomogram model involved several analytical steps. **(A)** The nomogram model was constructed based on MNS1 and HERC6. **(B)** The calibration curves of the nomogram in the GSE57338. **(C)** ROC curve for the diagnostic performance of the two candidate biomarkers (MNS1 and HERC6). **(D)** The DCA curves of nomogram in the GSE57338. **(E–G)** Calibration curve, ROC curve, and DCA decision curve for the nomogram in the external dataset GSE29819 of DCM. Abbreviations: ROC, receiver operating characteristic; AUC, the area under the curve; DCA, Decision curve analysis.

Logistic regression analysis was conducted for MNS1 and HERC6 to improve diagnostic and predictive capacities, revealing analogous expression trends (Supplementary File S4). Subsequently, we created a nomogram for these two genes as model genes, as illustrated in Figure 8A. The calibration curve analysis in this study revealed a Dxy value of 0.876, signifying a strong correlation between predicted and actual values. The Brier score was 0.097, indicating effective calibration. Additionally, the z-values and p-values from the Spiegelhalter Z-test are denoted as S:z and S:p, respectively, with the p-value exceeding 0.05, suggesting an acceptable model fit (Figure 8B). The area under the curve (AUC) of the two model genes and the nomogram was assessed by Receiver Operating Characteristic (ROC) analysis to ascertain their sensitivity and specificity in diagnosing CKD-associated DCM. The results indicated that the AUC values of the two model genes were above 0.84, but the nomogram demonstrated a superior AUC value relative to the two model genes, signifying its substantial diagnostic efficacy in identifying CKD-associated DCM (Figure 8C). Furthermore, decision curve analysis (DCA) was conducted to further investigate the predictive model, indicating that decision-making utilizing the nomogram may assist in detecting CKD-associated DCM (Figure 8D).

An external validation set from the GEO database, specifically the GSE29819 dataset of DCM patients, was utilized to validate the nomogram. The calibration curves, ROC curve analysis, and DCA of this column line graph demonstrated strong diagnostic performance for CKD-related DCM patients (Figures 8E–G).

### 3.8 Single gene GSEA

**FIGURE 9:**
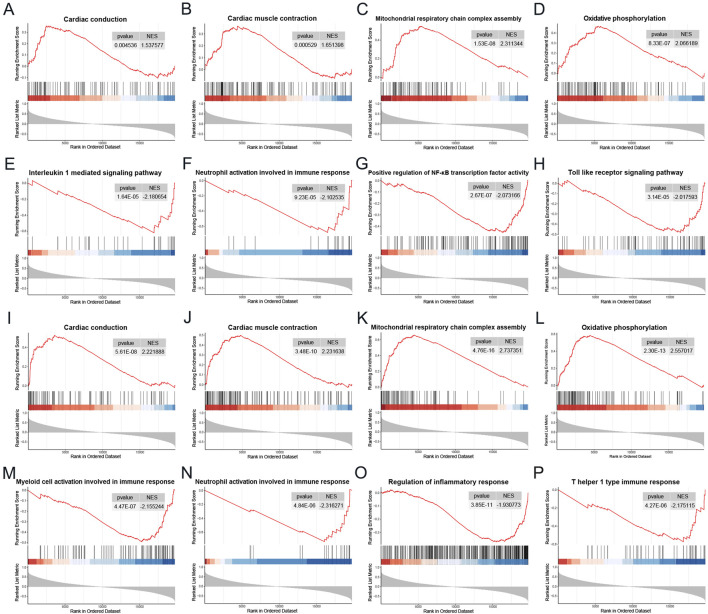
Single gene GSEA. **(A–H)** GSEA results of MNS1 in the DCM dataset GSE57338. **(I–P)** GSEA results of HERC6 in the DCM dataset GSE57338. Abbreviations: GSEA, Gene set enrichment analysis.

To elucidate the potential biological roles of the two genes in DCM, we conducted a single-gene Gene Set Enrichment Analysis (GSEA) using the Gene Ontology gene set. As illustrated in Figures 9A–H, the gene MNS1 is implicated in several crucial pathways, including “Cardiac conduction,” “Cardiac muscle contraction,” “Mitochondrial respiratory chain complex assembly,” and “Oxidative phosphorylation.” It is also involved in immune and inflammatory pathways such as the “Interleukin 1 mediated signaling pathway,” “Neutrophil activation involved in immune response,” “Positive regulation of NF-κB transcription factor activity,” and the “Toll-like receptor signaling pathway.” Similarly, the gene HERC6 shares regulatory pathways with MNS1, including “Cardiac conduction,” “Cardiac muscle contraction,” “Mitochondrial respiratory chain complex assembly,” and “Oxidative phosphorylation.” Additionally, HERC6 is engaged in processes related to the “Regulation of inflammatory response,” “Neutrophil activation involved in immune response,” “T helper 1 type immune response,” and “Myeloid cell activation involved in immune response,” as depicted in Figures 9I–P. These findings indicate that both genes may play significant roles in metabolic, immune, and inflammatory responses in the context of CKD-associated DCM.

### 3.9 Immune cell infiltration and correlation analysis

**FIGURE 10:**
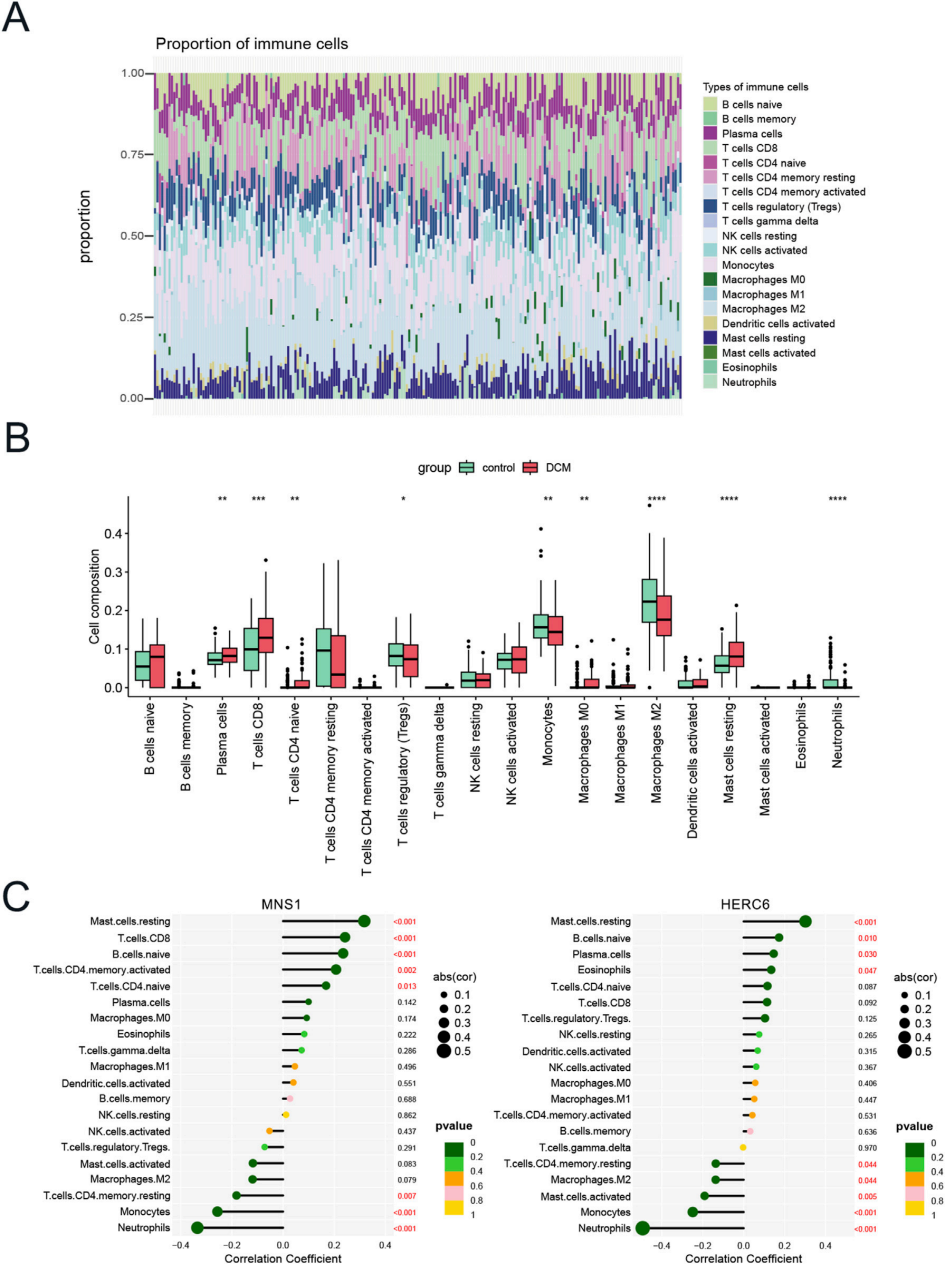
Immune cell infiltration and correlation analysis. **(A)** Bar graph of the percentage of 20 kinds of immune cells in each sample in GSE57338. **(B)** Box plot showing the comparison of 20 kinds of immune cells between DCM and control groups. **(C)** The correlation map represents the association of the immune cells with MNS1 and HERC6. Abbreviations: *p < 0.05, **p < 0.01, ***p < 0.001, ****p < 0.0001.

The enrichment analysis results revealed a significant correlation between the functional and pathway analysis of pathogenic genes associated with CKD and DCM and inflammatory and immunological processes. To further investigate this relationship, we utilized the CIBERSORT method to examine immune cell infiltration and its association with immunomodulatory and diagnostic biomarkers in CKD and DCM. Notably, the analysis identified a lack of expression in follicular helper T cells and resting dendritic cells, resulting in their exclusion from the study. Consequently, the bar graph (Figure 10A) illustrates the proportions of the remaining 20 immune cell types in each sample.

Figure 10B illustrates the immune cell profile of patients with DCM, revealing elevated levels of B cells naive, Plasma cells, T cells CD8, T cells CD4 naive, Macrophages M0, and Mast cells resting. In contrast, these patients exhibit decreased levels of memory-activated T cells CD4 memory activation, Macrophages M2, and Neutrophils. This differential pattern of immune cell infiltration suggests that various immune cells may play a regulatory role in the therapeutic management of DCM.

In our study, we performed a comprehensive analysis to explore the correlation between the expression of two model genes and the distribution of various invading immune cell types. As depicted in Figure 10C, the expression of MNS1 showed a substantial positive correlation with resting Mast cells, CD8 T cells, naive B cells, activated CD4 memory T cells, and naive CD4 T cells. Conversely, MNS1 exhibited a negative correlation with Neutrophils, Monocytes, and resting CD4 memory T cells. Similarly, the expression of HERC6 was positively correlated with resting Mast cells, naive B cells, Plasma cells, and Eosinophils. However, it demonstrated a negative correlation with Neutrophils, Monocytes, activated Mast cells, M2 Macrophages, and resting CD4 memory T cells. These findings indicate that immune cell infiltration in DCM samples undergoes significant alterations compared to the control group, and the expression levels of the two model genes are significantly correlated with these changes in immune cell infiltration.

### 3.10 Distribution and expression of hub genes in the heart

**FIGURE 11:**
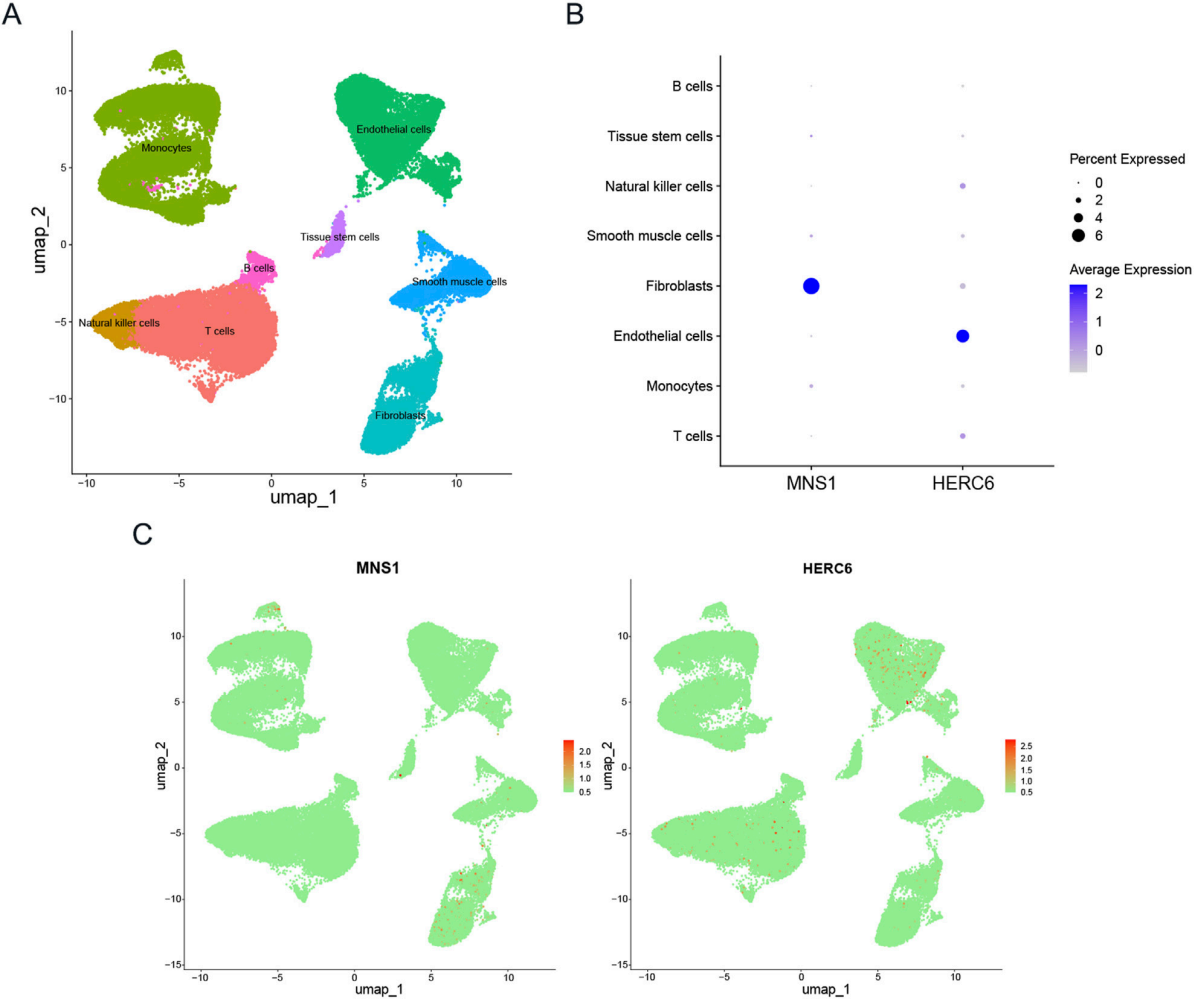
Expression profiles of model genes in single cells. **(A)** Cellular subtypes of dilated cardiomyopathy. **(B,C)** Bubble plot and scatter plots of the expression of the 2 module genes.

Single-cell data from GSE145154, sourced from publicly accessible DCM single-cell RNA sequencing resources, facilitated the identification of 21 distinct cell clusters. These clusters were primarily composed of T cells, monocytes, endothelial cells, smooth muscle cells, natural killer cells, fibroblasts, tissue stem cells, and B cells, as illustrated in Figure 11A(Huang et al., 2024). Furthermore, the expression levels of MNS1 and HERC6 across these various cell types are presented in Figures 11B,C, respectively.

## 4 Discussion

The incidence of heart failure and sudden cardiac mortality escalates with the advancement of chronic kidney disease, which may be linked to the heightened prevalence of CKD-associated DCM as renal function deteriorates (Law et al., 2023). Nonetheless, the fundamental mechanisms underlying the development of CKD-associated DCM remain little comprehended, and considerable ambiguity exists in its diagnosis. This study thoroughly examined CKD-associated DCM utilizing various bioinformatics tools and analytical approaches. The study sought to elucidate the potential relationship between CKD and DCM, investigate probable shared pathological mechanisms, and identify viable biomarkers for diagnosing CKD-associated DCM.

In this study, differential gene expression analysis and WGCNA identified 47 common differentially expressed genes. Subsequent GO and KEGG analyses revealed that these genes are predominantly enriched in pathways related to inflammation and immune response. This enrichment suggests that immunological and inflammatory processes play a significant regulatory role in the pathogenesis of CKD and DCM.

In the development of a diagnostic model for predicting DCM onset in chronic kidney disease patients, MNS1 and HERC6 emerged as significant model genes. Single-cell sequencing analysis revealed distinct cellular distribution patterns, with MNS1 showing enrichment in fibroblasts and HERC6 predominantly expressing in endothelial cells within cardiac tissues. Correlation analyses demonstrated that these biomarkers exhibited significant positive associations with resting mast cells and naive B cells while displaying inverse relationships with monocytes and neutrophils. These differential correlation patterns with immune cell populations suggest that both genes may contribute to DCM pathogenesis through immune infiltration-mediated mechanisms.

MNS1 (meiosis-specific nuclear structural protein 1) is a protein-coding gene primarily associated with the function of motile cilia and the assembly of sperm flagella (Maraval et al., 2025).Existing research indicates that abnormalities in MNS1 may increase the risk of congenital cardiac disease through ciliopathies affecting active cilia, such as laterality defects. (Ta-Shma et al., 2018; Perrot and Rickert-Sperling, 2024). Notably, in 2022, Jiang et al. discovered that high expression of the MNS1 gene is involved in the metabolism of bile acids, fatty acids, and heme, which in turn influences the progression of heart failure (HF) and demonstrates significant diagnostic efficacy for HF (Jiang et al., 2022). In 2023, Duan et al. further supported the role of MNS1 in the early diagnosis of HF (Duan et al., 2023). Additionally, MNS1 has been shown to be highly expressed in patients with ischemic cardiomyopathy (Zheng et al., 2023). Collectively, these findings suggest that MNS1 may play a significant regulatory role in cardiac diseases; however, its specific relationship with DCM remains unclear. GSEA analysis suggests that MNS1 is implicated in the regulation of cardiac conduction, myocardial contraction, oxidative phosphorylation, and the inhibition of immune and inflammatory processes. In this study, MNS1 expression was significantly elevated in patients with DCM compared to healthy controls. Additionally, MNS1 showed a strong positive correlation with resting mast cells, CD8 T cells, and naive B cells, while displaying a negative correlation with monocytes and neutrophils. These findings imply that MNS1 may contribute to the pathogenesis of DCM through modulation of these immune cell populations; nonetheless, further research is necessary to validate these findings.

Members of the HERC subfamily, as ubiquitin E3 ligases, are distinguished by the presence of a HECT domain and one or more RCC1-like domains (RLDs) serving as regulatory elements. The subfamily can be further categorized into two subclasses: large HERC (comprising HERC1 and HERC2) and small HERC (containing HERC3 through HERC6) (Rotin and Kumar, 2009). As a member of the HERC family, HERC6 is significantly expressed in testicular tissue and fetal brain, and it is also expressed in cardiac tissue, where it is involved in various cellular activities, including cell proliferation, cell migration, and neurodevelopment (Zhang et al., 2024). Recent studies on systemic lupus erythematosus have revealed the key role of HERC6 in apoptosis and inflammation regulation (Cao et al., 2022). HERC6 is a pivotal effector of vertebrate innate immunity with substantial antiviral efficacy (Paparisto et al., 2018; Paparisto, 2023). Nonetheless, its function in cardiovascular disease remains inadequately defined, necessitating further studies to understand its precise effects and processes in DCM (Zhong et al., 2024). The single-gene GSEA analysis of HERC6 in this study indicates that this gene may participate in the regulation of the disease in DCM patients by inhibiting the activation of myeloid cells and neutrophils involved in immune responses, negatively regulating inflammatory responses, and promoting biological processes such as cardiac contraction and oxidative phosphorylation. In terms of immune infiltration analysis, HERC6 exhibited a positive correlation with mast cells resting and a negative correlation with the infiltration of monocytes and neutrophils. Building on the aforementioned points, we hypothesize that HERC6 may serve a dual function in regulating immune responses. Firstly, it could modulate the behavior of mast cells and other immune cells, thereby limiting immune activation and minimizing harmful inflammatory reactions. Such regulation may be essential for protecting the heart from immune-induced injury. Secondly, HERC6 may suppress immune and inflammatory pathways by promoting the ubiquitination and degradation of specific pro-inflammatory cytokines or their receptors.Through these mechanisms, HERC6 could serve as a critical modulator in maintaining immune homeostasis and preventing excessive inflammation. Furthermore, the expression of HERC6 may be linked to oxidative phosphorylation in heart contractile function and energy metabolism, indicating its potential significance in sustaining cardiac function. Nonetheless, these conjectures necessitate additional experimental verification to elucidate the precise mechanism of HERC6’s involvement in DCM. Future research should concentrate on the functional validation of HERC6 in heart disorders and assess its viability as a therapeutic target.

To advance novel diagnostic tools and therapeutic approaches for CKD-associated DCM, a comprehensive investigation of MNS1 and HERC6 expression patterns and functional roles is essential. Given their potential significance, these molecular markers are emerging as promising diagnostic and therapeutic targets. To validate their clinical utility, we constructed a diagnostic nomogram and evaluated its performance using an external dataset. The nomogram’s diagnostic accuracy was rigorously assessed through multiple analytical methods, including ROC curve analysis, decision curve analysis, and calibration curve analysis, which collectively demonstrated its efficacy in identifying patients with CKD-associated DCM.

In addressing CKD-associated DCM, we have employed the CMAP database to evaluate various small molecule drugs with possible therapeutic benefits. Among them, Droxinotat, a histone deacetylase inhibitor, was developed by Reed et al. (Schimmer et al., 2006; Mawji et al., 2007; Wood et al., 2010), and the compound was able to effectively inhibit the enzymatic activities of HDAC3, HDAC6, and HDAC8, and showed significant anticancer potential (Kerr et al., 2012). HDAC3 is a crucial regulator of cardiomyocyte proliferation during heart development (Reichert et al., 2012). Furthermore, a study using a mouse model of dilated cardiomyopathy highlighted the cardioprotective efficacy of HDAC6 inhibitors, suggesting their potential as a therapeutic approach for cardiomyopathy and various forms of heart failure (Yang et al., 2022). Additionally, HDAC8 inhibitors have been shown to reduce ventricular hypertrophy and fibrosis, as well as alleviate symptoms of heart failure (Zhao et al., 2021; 2022). These findings support the feasibility of Droxinotat as a potential therapeutic agent for CKD-associated DCM. In addition to Droxinotat, Anisomycin, a selective activator of the p38 MAPK pathway, has demonstrated cardioprotective properties (Zhao et al., 2001). Another promising compound is Withaferin A (WFA), a bioactive withanolide extracted from the medicinal plant Withania somnifera. WFA exhibits antitumor and immunomodulatory activities against various cancer cells (Yan et al., 2018). At low doses, WFA exerts cardioprotective effects by upregulating the anti-apoptotic mitochondrial pathway in an AMPK-dependent manner (Guo et al., 2019). An experimental study on Withaferin A revealed its ability to reduce type I collagen expression *in vitro* and impede the progression of cardiac fibrosis *in vivo* (Challa et al., 2012). Furthermore, WFA offers renal protection by reducing inflammation and macrophage signaling in obstructed kidneys, as well as inhibiting the development of fibrosis and TGF-β signaling (Peddakkulappagari et al., 2019). Collectively, these drugs hold significant potential for addressing CKD-related DCM, providing novel insights for the advancement of future therapeutic strategies.To further validate the therapeutic potential of these candidate compounds in the context of CKD-associated DCM, we plan to conduct follow-up experimental studies. These will include *in vitro* assays using co-culture systems of cardiomyocytes and renal epithelial cells to assess the compounds’ effects on fibrosis, inflammation, and apoptosis-related pathways. Additionally, we aim to establish *in vivo* models of CKD-induced cardiomyopathy to evaluate the efficacy and safety profiles of Droxinostat, Withaferin A, and other top-ranked candidates. These studies will provide essential functional evidence to support their clinical translational potential.

This study, despite its contributions, has several limitations that warrant consideration. Firstly, CKD is a broad term encompassing a wide range of conditions, and this study did not investigate specific types of CKD, which may limit the applicability of the findings to particular subtypes. Secondly, although machine learning and WGCNA are widely used analytical methods, their results can vary significantly depending on the dataset employed, potentially affecting the consistency and reliability of the outcomes. Finally, to improve the applicability of our findings to clinical practice, it is essential to conduct both *in vivo* and *in vitro* validation studies.

## 5 Summary

In our study, we evaluated the transcriptomic data of patients with CKD and DCM to identify DEGs and key module genes shared by both diseases. Subsequent analyses included functional enrichment, WGCNA, connectivity map analysis, immune cell infiltration, and single-cell sequencing. Our findings suggest the presence of a potential comorbidity mechanism between CKD and DCM, possibly mediated by model genes. This research contributes to understanding the molecular mechanisms underlying CKD and DCM.

In conclusion, this study proposes a potential diagnostic and assessment approach for patients with CKD-associated DCM. By exploring the intersection of CKD and DCM, we suggest that future screening for CKD-associated DCM could involve detecting immune or inflammatory factors, as well as employing genetic testing or emerging advanced technologies for precise diagnosis (Liu et al., 2023a; Baysoy et al., 2024; Fan et al., 2024).Furthermore, we propose the possibility of targeted therapy for patients with CKD and DCM based on the gene loci identified in this study. However, these theoretical insights require further validation through clinical trials.

## Data Availability

Publicly available datasets were analyzed in this study. This data can be found here: Gene Expression Omnibus(https://www.ncbi.nlm.nih.gov/geo/) The datasets include one gene expression profile for CKD (GSE104954), two gene expression profiles for DCM (GSE57338, GSE29819), and one single-cell RNA sequencing dataset for DCM (GSE145154).
