# Supplementary figures and images for: Identification of biomarkers for the diagnosis of chronic kidney disease (CKD) with dilated cardiomyopathy (DCM) by bioinformatics analysis and machine learning
Source: Front Genet. 2025 May 30;16:1562891. doi: 10.3389/fgene.2025.1562891 (PMC12162942; doi:10.3389/fgene.2025.1562891)

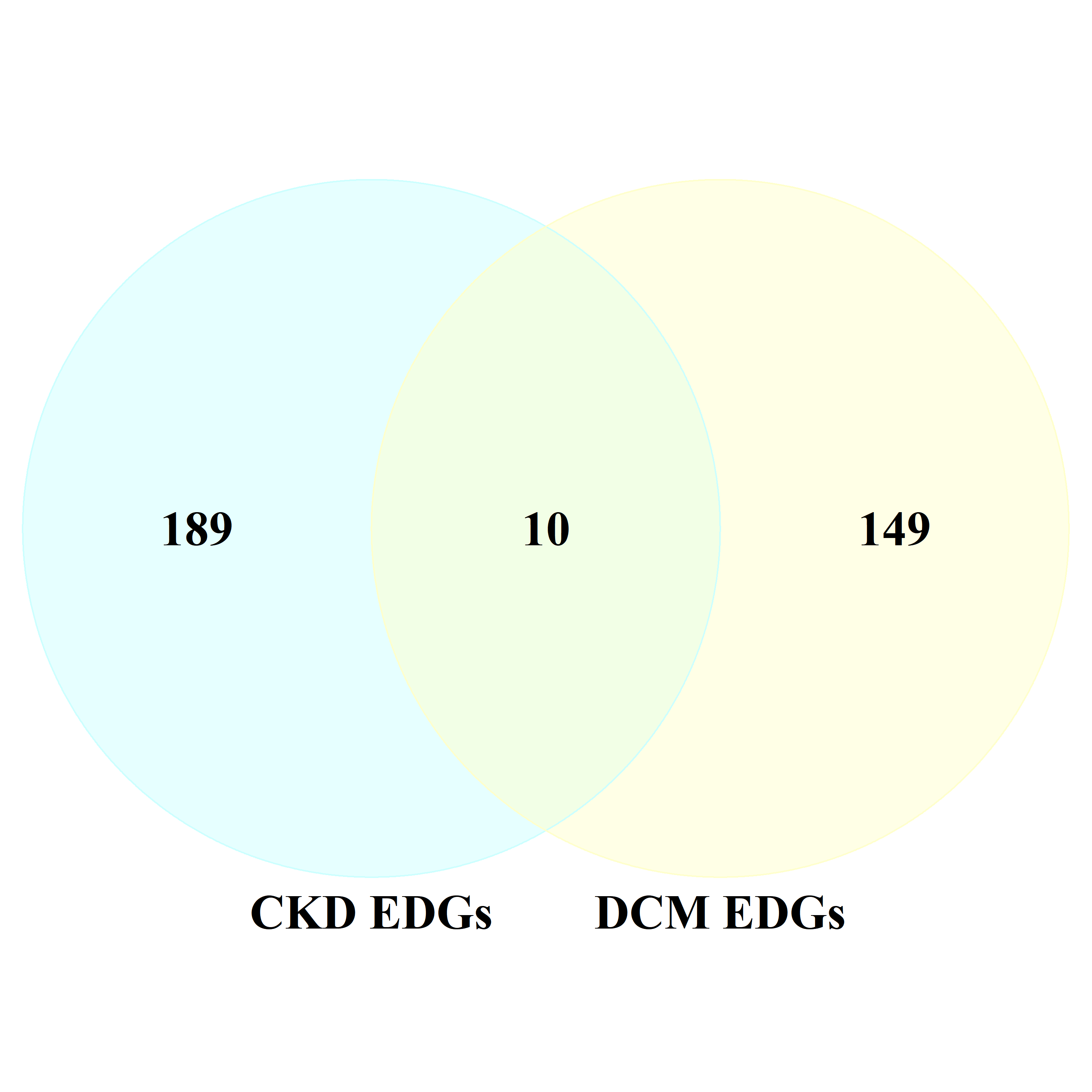

Supplement: Supplementary file 1 [file DataSheet3.zip › co-regulated DEGs/Co-downregulated genes/Venn diagram of co-downregulated genes.png]

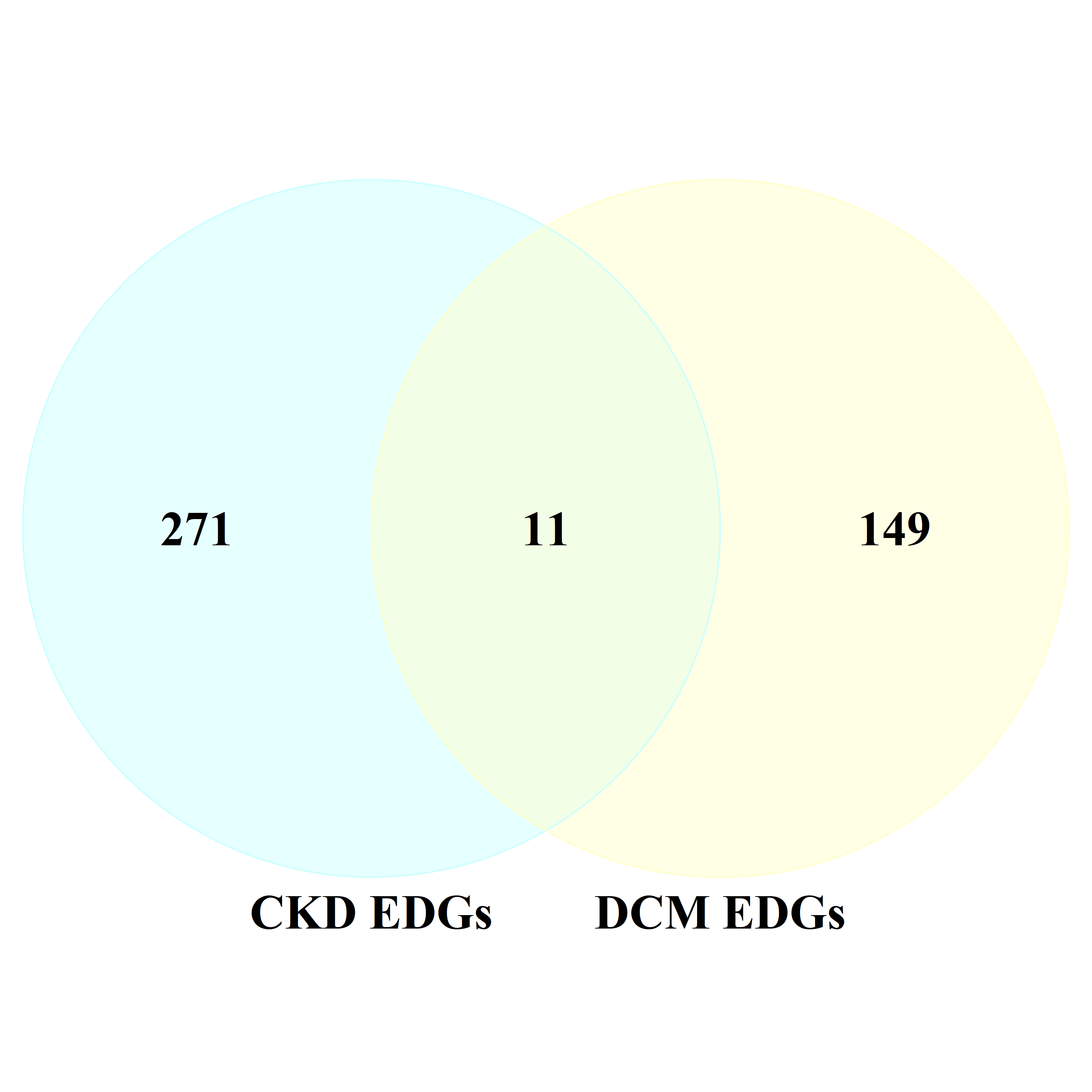

Supplement: Supplementary file 1 [file DataSheet3.zip › co-regulated DEGs/Co-upregulated genes/Venn diagram of co-upregulated genes.png]

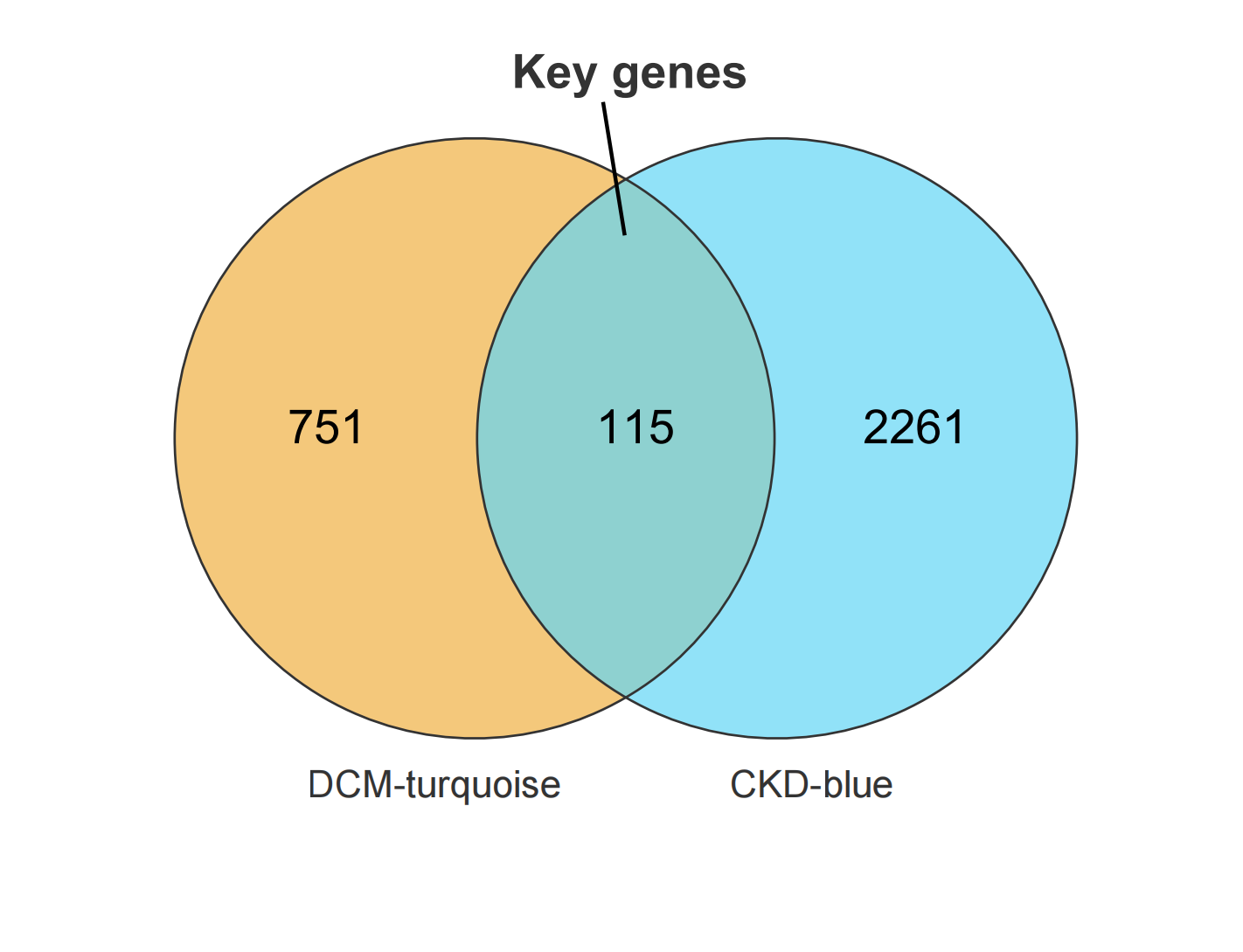

Supplement: Supplementary file 3 [file Image1.tif]

HERC6 Expression vs Type

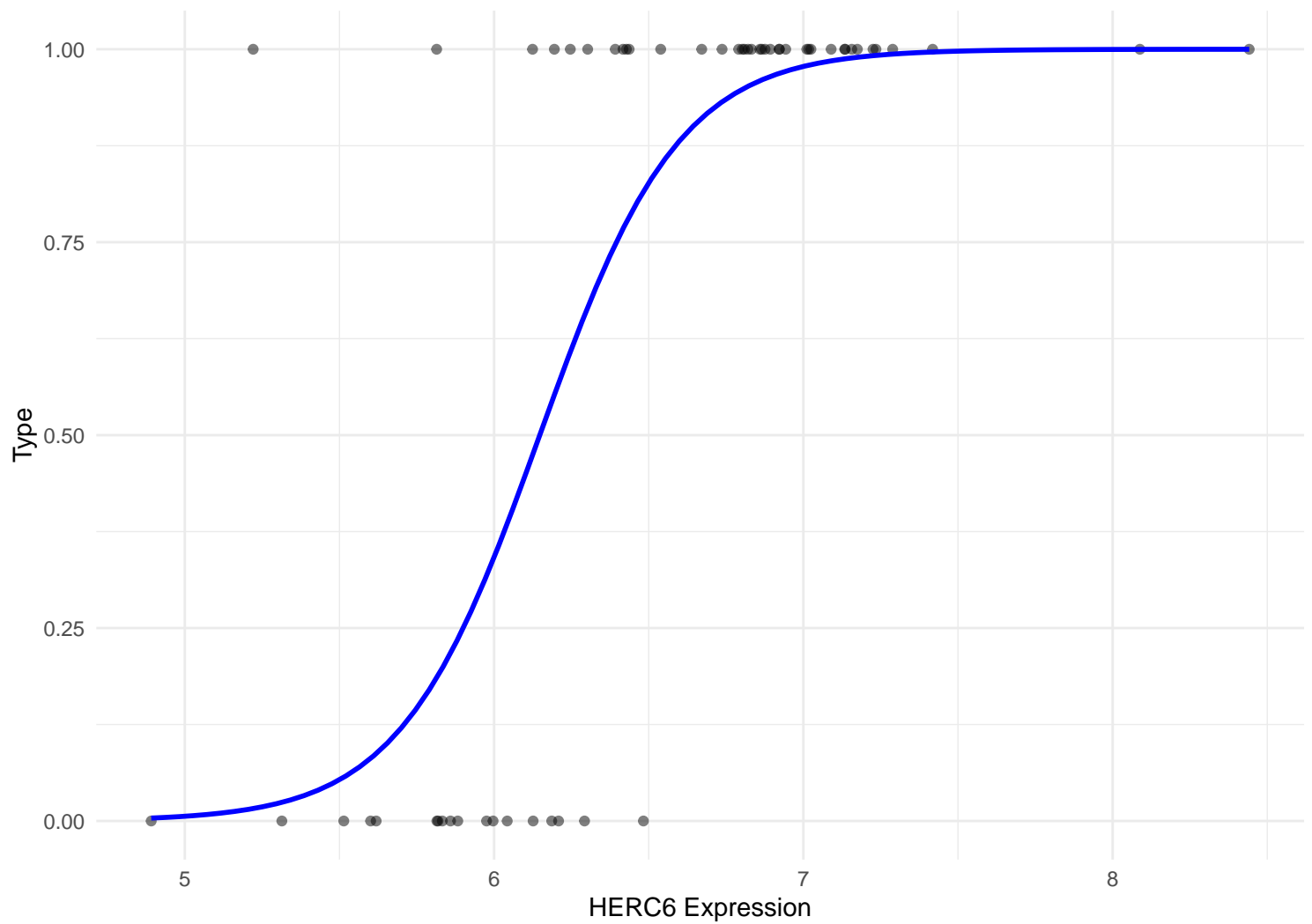

Supplement: Supplementary file 4 [file DataSheet2.zip › Logistic Regression Trend/CKD/Expression trend chart of HERC6.pdf]

MNS1 Expression vs Type

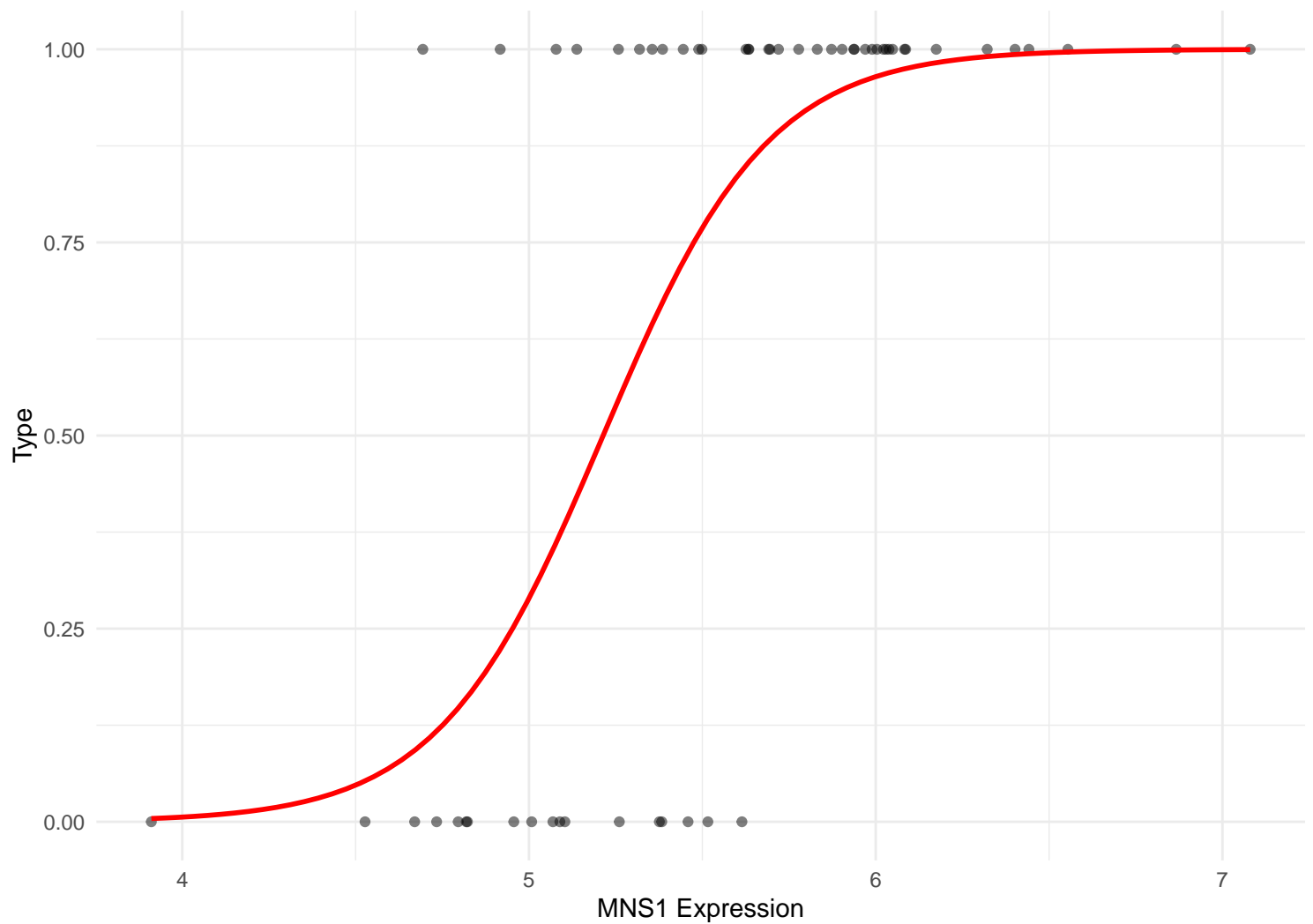

Supplement: Supplementary file 4 [file DataSheet2.zip › Logistic Regression Trend/CKD/Expression trend chart of MNS1.pdf]

HERC6 Expression vs Type

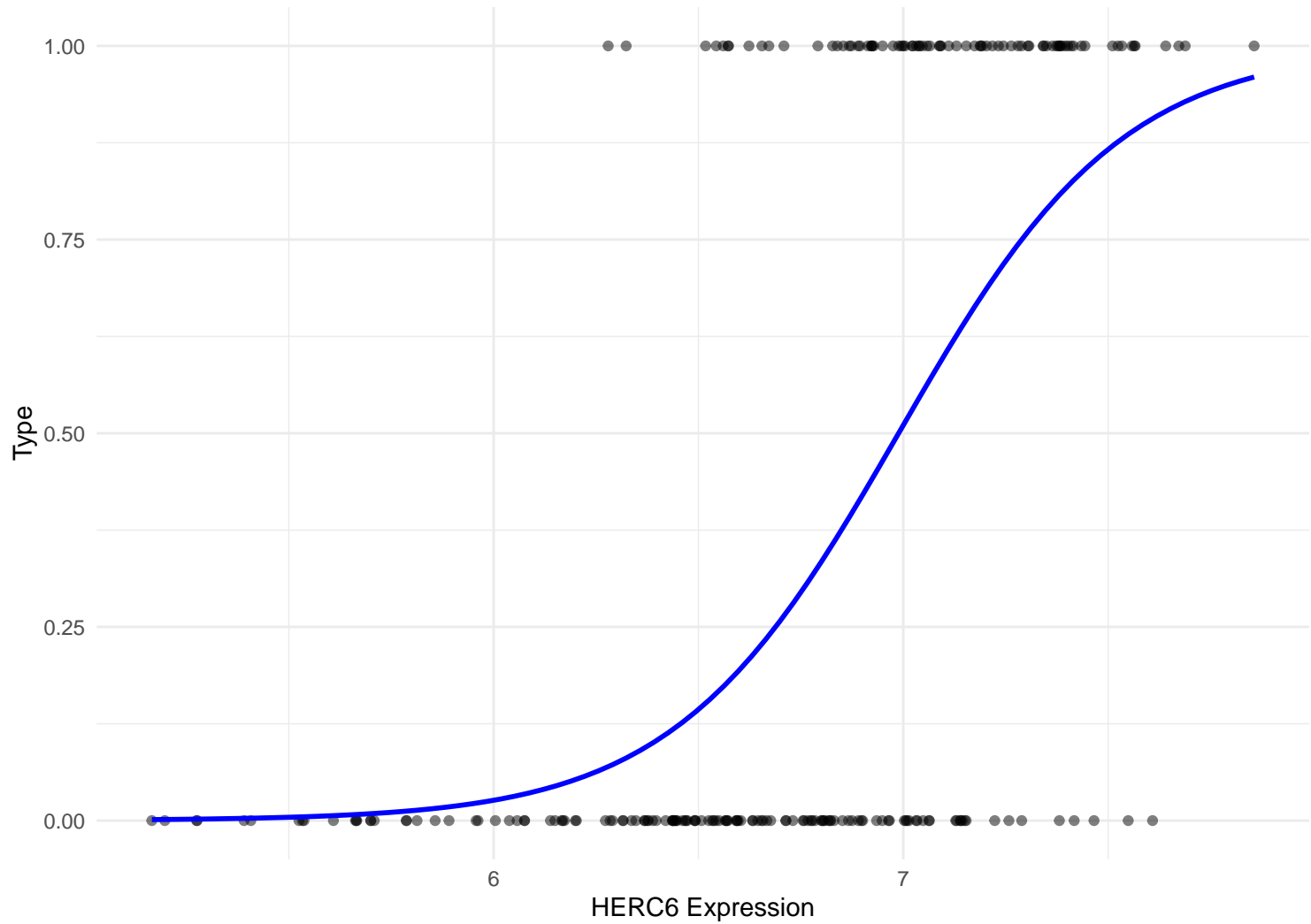

Supplement: Supplementary file 4 [file DataSheet2.zip › Logistic Regression Trend/DCM/Expression trend chart of HERC6.pdf]

MNS1 Expression vs Type

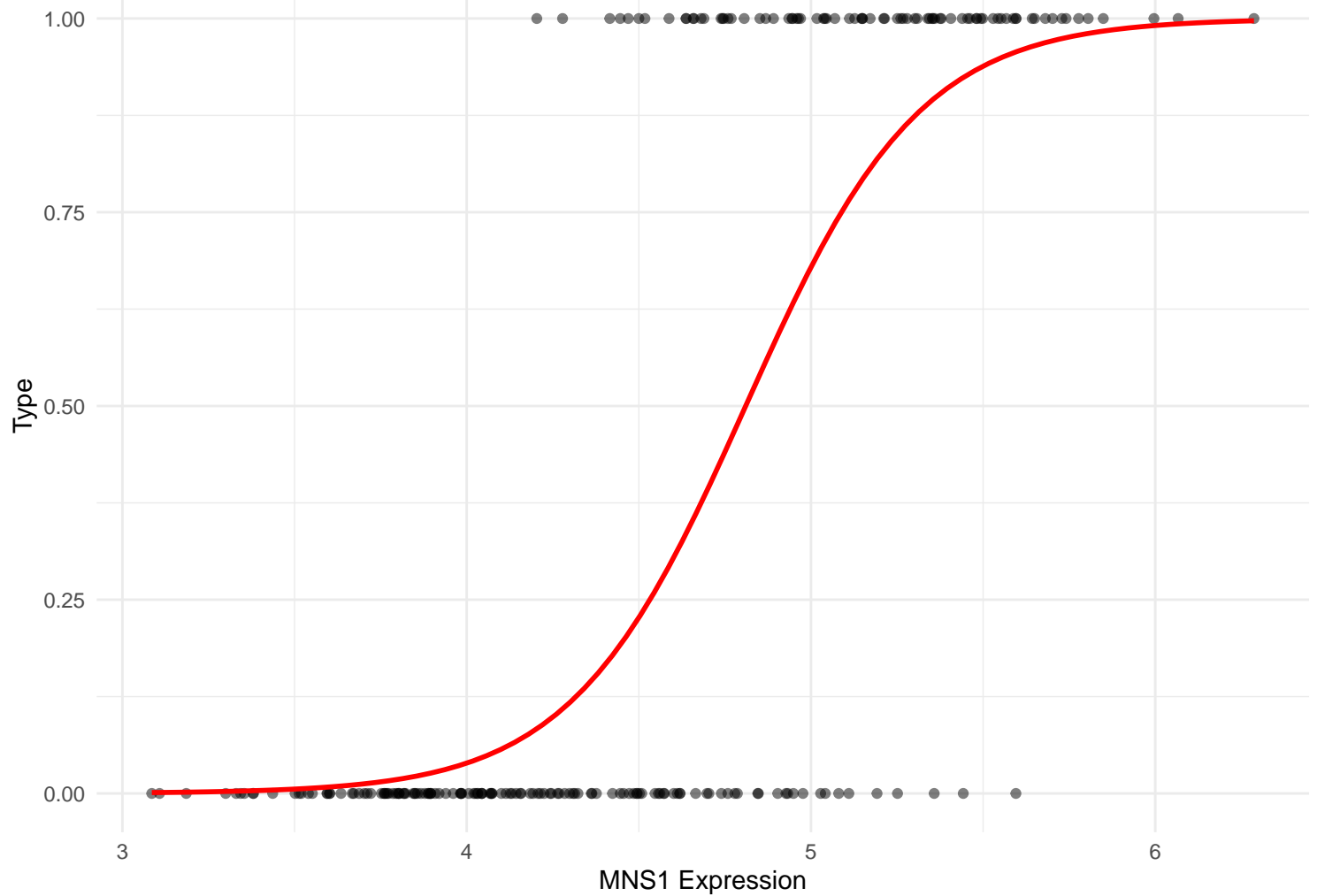

Supplement: Supplementary file 4 [file DataSheet2.zip › Logistic Regression Trend/DCM/Expression trend chart of MNS1.pdf]
